# Analysis of COVID-19-Related RT-qPCR Test Results in Hungary: Epidemiology, Diagnostics, and Clinical Outcome

**DOI:** 10.3389/fmed.2020.625673

**Published:** 2021-01-26

**Authors:** Katalin Gombos, Mária Földi, Szabolcs Kiss, Róbert Herczeg, Attila Gyenesei, Lili Geiger, Dávid Csabai, Krisztina Futács, Tamás Nagy, Attila Miseta, Balázs Antal Somogyi, Péter Hegyi, Andrea Szentesi

**Affiliations:** ^1^Department of Laboratory Medicine, Clinical Center, Medical School, University of Pécs, Pécs, Hungary; ^2^Institute for Translational Medicine, Medical School, University of Pécs, Pécs, Hungary; ^3^Szentágothai Research Centre, University of Pécs, Pécs, Hungary; ^4^Centre for Translational Medicine, Department of Medicine, University of Szeged, Szeged, Hungary; ^5^Bioinformatics Research Group, Genomics and Bioinformatics Core Facility, Szentágothai Research Centre, University of Pécs, Pécs, Hungary; ^6^Clinical Research Centre, Medical University of Bialystok, Bialystok, Poland; ^7^Neurobiology of Stress Research Group, Szentágothai Research Centre, University of Pécs, Pécs, Hungary; ^8^National Virology Laboratory, Szentágothai Research Centre, University of Pécs, Pécs, Hungary

**Keywords:** COVID-19, SARS-CoV-2, PCR diagnostics, testing, epidemiology, surveillance

## Abstract

**Background:** Effective testing is an essential tool for controlling COVID-19. We aimed to analyse the data from first-wave PCR test results in Hungary's Southern Transdanubian region to improve testing strategies.

**Methods:** We performed a retrospective analysis of all suspected COVID-19 cases between 17 March and 8 May 2020, collecting epidemiological, demographic, clinical and outcome data (ICU admission and mortality) with RT-qPCR test results. Descriptive and comparative statistical analyses were conducted.

**Results:** Eighty-six infections were confirmed among 3,657 tested patients. There was no difference between the positive and negative cases in age and sex distribution; however, ICU admission (8.1 vs. 3.1%, *p* = 0.006) and in-hospital mortality (4.7 vs. 1.6%, *p* = 0.062) were more frequent among positive cases. Importantly, none of the initially asymptomatic patients (*n* = 20) required ICU admission, and all survived. In almost all cases, if the first test was negative, second and third tests were performed with a 48-h delay for careful monitoring of disease development. However, the positive hit rate decreased dramatically with the second and third tests compared to the first (0.3 vs. 2.1%, OR = 0.155 [0.053–0.350]). Higher E-gene copy numbers were associated with a longer period of PCR positivity.

**Conclusion:** In our immunologically naïve suspected COVID-19 population, coronavirus infection increased the need for intensive care and mortality by 3–4 times. In the event of the exponential phase of the pandemic involving a bottleneck in testing capacity, a second or third test should be reconsidered to diagnose more coronavirus infections.

## Introduction

In December 2019, a new strain of human coronavirus, the Severe Acute Respiratory Syndrome Coronavirus 2 (SARS-CoV-2), emerged, causing the Coronavirus Disease 2019 (COVID-19) (1). From the beginning of the pandemic to 1 November 2020, more than 46 million individuals have been infected and more than 1.2 million have died from the infection in 216 affected countries (2). Incidence is still rising on a global scale, and countries are well into the second or third wave of the pandemic.

Due to the possibility of rapid human-to-human transmission and lack of specific therapy, fast, and reliable diagnostic tests are essential. Timely and rapid testing prevents the spread of the virus and optimizes infection control measures. It enables early case identification, isolation of cases and comprehensive contact tracing (3). By the end of the first wave, it became clear that countries that test more have lower mortality rates (4–6).

To identify active cases, nucleic acid amplification tests (NAAT), such as the quantitative reverse-transcription polymerase chain reaction (RT-qPCR) test, are the gold standard (7, 8). During the pandemic, many countries have faced difficulties in maintaining effective screening due to limited access to laboratory equipment and commercial consumables for PCR. Although these tests are now more widely available, rapid increase in testing requirements in different loci throughout the pandemic can interfere with testing capacity (9).

If transmission intensity exceeds testing capacity, countries need to prioritize who will be tested. There are international recommendations on the prioritization of testing among new suspected cases. As regards retesting, protocols are not evidence-based, and they differ among countries and hospitals (10). Retesting of initially negative cases and follow-up testing of positive cases need to be rationalized.

In addition to rationalizing the testing strategy, the allocation of scarce resources among COVID-19 cases is vital. Timely shifting of resources to higher risk groups is an option. Therefore, identification of early prognostic factors can help clinicians' decisions. Some risk factors, such as older age or obesity and the presence of comorbidities, have already been identified (11, 12). However, the role of several other factors is still unclear (13). For instance, our understanding of differences between symptomatic and asymptomatic cases is limited.

We aimed to analyse the data of first-wave PCR test results in Hungary's Southern Transdanubian region to aid in decision-making on the necessity and timing of testing and retesting, especially when testing capacity is limited.

## Materials and Methods

We performed a retrospective analysis of all suspected COVID-19 cases between 17 March and 8 May 2020. We used the definition of case according to the WHO interim guidance (14). Diagnostic PCR tests were performed by the accredited Department of Laboratory Medicine of the Medical School at the University of Pécs. This center is responsible for PCR testing of all samples in Hungary's Southern Transdanubian region.

### Patients Involved in the Analyses

Subjects enrolled in our epidemiological analysis were identified for SARS-CoV-2 PCR testing by the regional offices of Hungary's National Public Health and Medical Officer Service, 29 health care providers, including hospital and clinical sites, and 258 general practices in the Southern Transdanubian region (including four counties: Somogy, Tolna, Baranya, and Zala). All included cases were tested with PCR, and COVID-19 was diagnosed based on WHO interim guidance.

Testing criteria covered epidemiological and/or clinical indication (presence of symptoms listed on a questionnaire provided by our laboratory). Health care personnel who were contacts of confirmed COVID-19 patients were also enrolled.

*Epidemiological indication for testing* was defined as (1) close contact with a confirmed COVID-19 case and (2) travel from a COVID-19-affected area within 14 days to symptom onset. Epidemiological risk assessment and contact tracing were carried out by regional and national public health officers who did interviews about exposure, travel history and symptoms, identified contacts of confirmed COVID-19 patients, ordered isolation and monitored symptom development.

*Clinical indication for testing* included (1) presence of fever and/or upper and lower respiratory symptoms, (2) cough, (3) chest discomfort or pain, (4) shortness of breath or breathing difficulties, and (5) gastrointestinal symptoms, including abdominal discomfort, nausea, vomiting, and diarrhea. General practitioners and clinicians indicated testing of symptomatic patients.

The algorithm for SARS-CoV-2 PCR testing is summarized in Supplementary Figure 1.

### Outcomes and Data Collections

Participants' medical records were analyzed, and the following epidemiological, demographic, clinical, and outcome data were collected: reason for testing, date of sampling, age, gender, presence of symptoms, viral excretion, ICU admission, and mortality. Mortality data refer to the hospitalized period (in-hospital mortality). An assessment of viral excretion is detailed below and in Supplementary Document 1.

### Sampling

Samples were taken by health care professionals at the National Emergency Service or local health care providers. Specimens were collected from the lower respiratory tract (with a tracheal sputum) among hospitalized and ventilated cases and from the upper respiratory tract (with a nasopharyngeal or oropharyngeal swab) in all other participants. All specimens were labeled with the patient (or contact) name, date of birth, specimen type, and date, and time of collection.

Sample collection tubes were individually packaged in a sterile double wall plastic bag and transferred to the laboratory at 4°C for nucleic acid extraction. Nucleic acid was extracted from 200 μl specimens either manually or with the MagNaPure 96 automated nucleic acid extraction system (Roche, Mannheim, Germany). PCR amplification was carried out in LightCycler 480 and Cobas Z 480 PCR systems. Fluorescence data were converted into concentrations using a standard curve and analyzed accordingly. The test was considered positive if the sample was positive for at least two genes in the fortieth PCR cycle (cycle threshold/Ct value = 40) (15). Sample processing and PCR amplification are detailed in Supplementary Document 1.

### Statistical Analyses

All of the statistical analyses were performed using the R statistical environment, R Core Team v3.6.1 (16). A *p*-value < 0.05 was considered as statistically significant. For age, we calculated the median and the range. The Wilcoxon Rank Sum and Signed Rank Tests were used to compare the age between the negative and positive cases. For sex, the presence of symptoms, ICU admission and mortality odds ratios (OR) with the corresponding 95% confidence interval (CI) were calculated with the odds ratio function from the epitools package for R (17). The Wilcoxon Rank Sum and Signed Rank Tests were used for viral excretion (copy number of the E-gene).

### Ethical Issues

In this study, data were collected retrospectively and analyzed in compliance with ethical requirements. Ethical approval was granted by the National Centre for Public Health (20800-6/2020/EÜIG).

## Results

**Figure 1:**
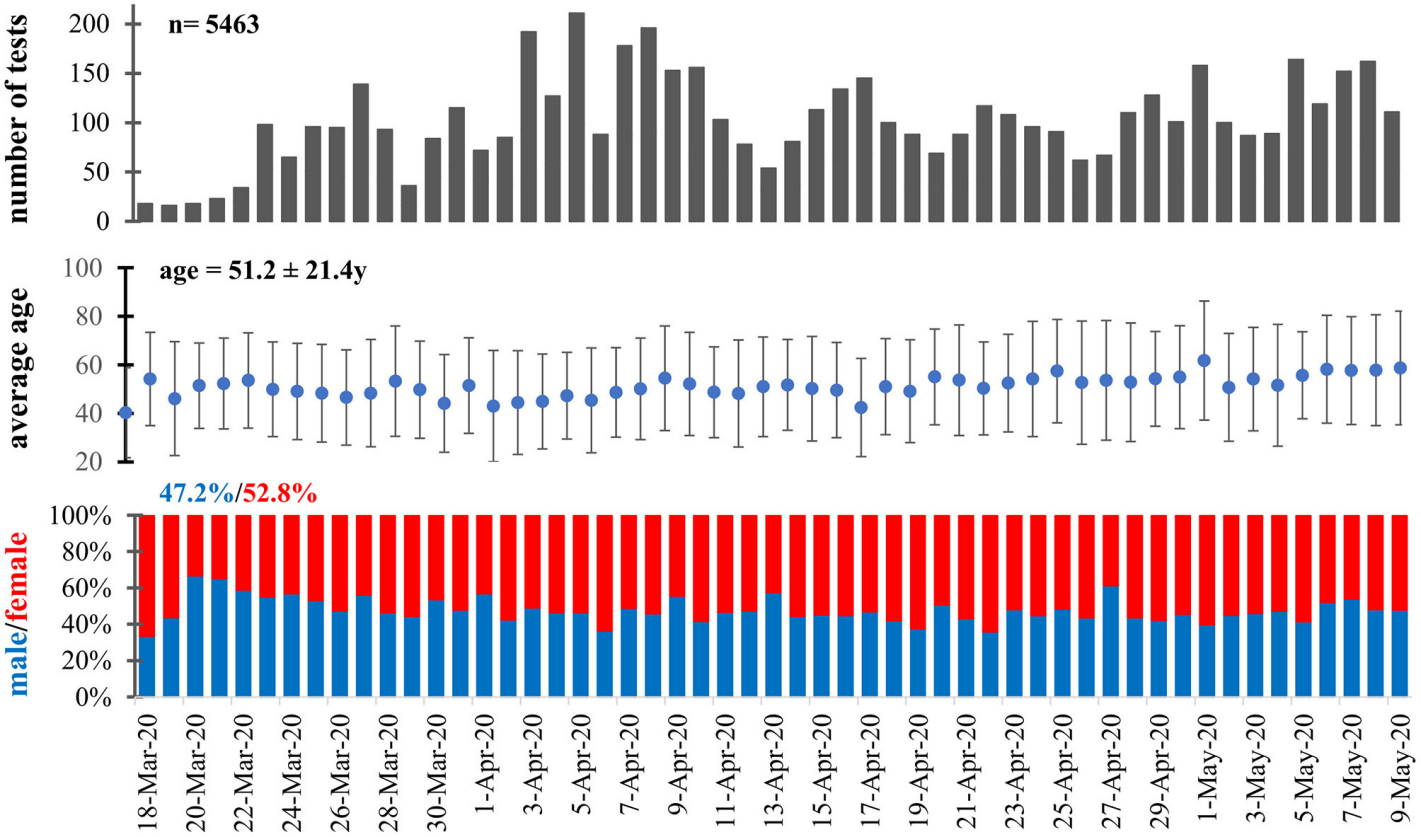
Age and sex distribution of the study population during the observation period.

Between 17 March and 8 May 2020, 3,657 people with suspected SARS-CoV-2 infection were tested in Hungary's Southern Transdanubian region, which represents 41.67 per thousand of the resident population living there (18). Among individuals with suspected COVID-19, a total of 5,463 tests were performed for 3,657 people, and 86 infections (2.35% of all participants and 1.57% of the total tests) were confirmed positive. The number of tests performed showed a steady increase in the first week and then relative stability until the end of the observation period. The median age of the individuals tested was found to be 52 years (range 0–98), and the proportion of male participants was 47.2%. During the study period, the mean age and sex distribution also showed relative constancy (Figure 1).

### Dynamics of the COVID-19 Pandemic

**Figure 2:**
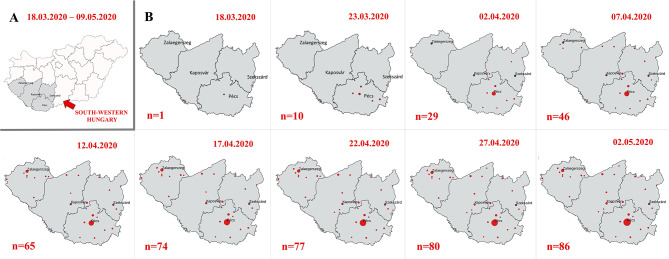
Incident cases representing the dynamics of the pandemic spreading in 5-day intervals. **(A)** Hungary and **(B)** south-western Hungary.

The first case confirmed by PCR was found in Pécs on 18 March 2020, along with some symptomatic cases and contacts during the following 5 days. The last new positive case was diagnosed on 2 May. PCR-confirmed SARS-CoV-2-infected cases are plotted on the map of Hungary's Southern Transdanubian region. The increase of incident cases representing the dynamics of the pandemic spreading can be followed by 5-day intervals (Figure 2). The mapping methodology is specified in Supplementary Document 1. Most confirmed cases were identified in large cities in the region, e.g., Pécs, while only a few cases were found in rural areas there.

### Clinical Characteristics of Coronavirus Infection

**Figure 3:**
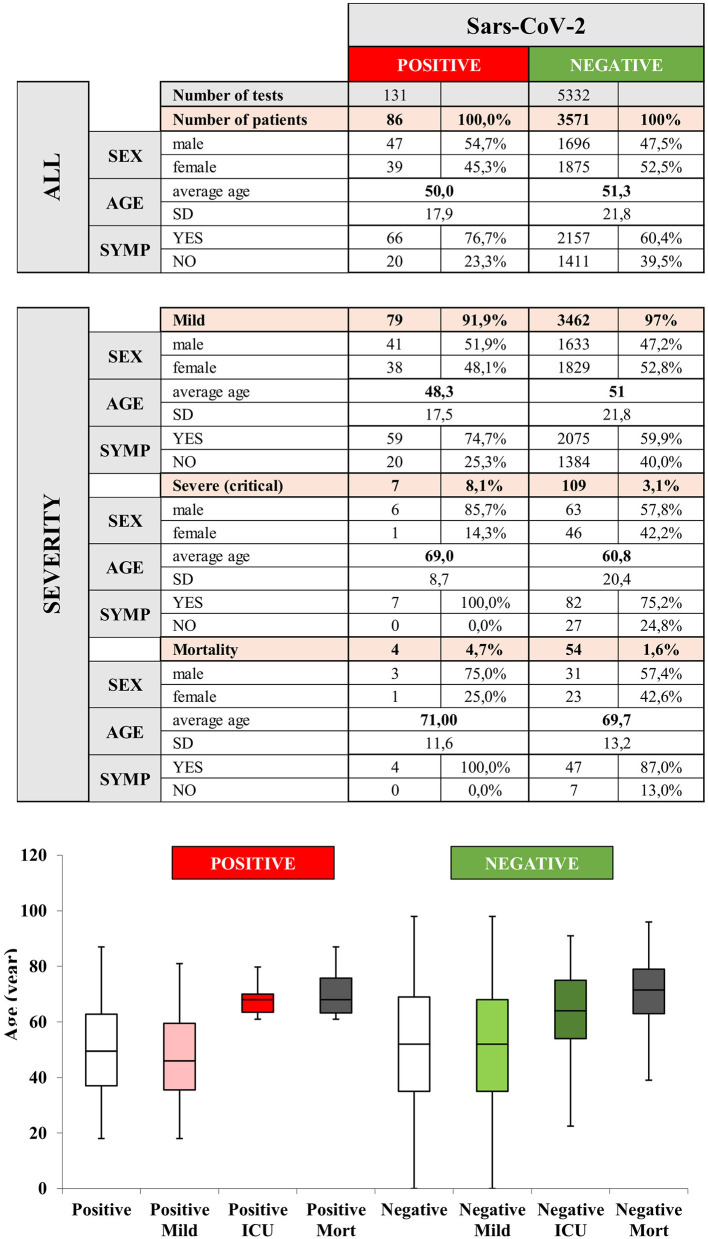
Epidemiology and clinical outcome of the study population and comparison of negative and positive cases confirmed by polymerase chain reaction (PCR). SYMP, symptoms; SD, standard deviation; ICU, intensive care unit; Mort, mortality.

Comparative analyses of the SARS-CoV-2 PCR test-positive and test-negative subpopulations were performed for age, sex, presence of symptoms, ICU admission and mortality (Figure 3). There was no statistical difference between positive and negative cases in terms of age at diagnosis (50.0 ± 17.9 vs. 51.3 ± 21.8, respectively; W = 162,302, *p* = 0.353), but a slightly lower proportion of women was observed among the confirmed cases (46.5 vs. 53.4%, OR = 0.787 [0.510–1.209], *p* = 0.274). Among suspected COVID-19 cases, the proportion of symptomatic patients was higher in those with a positive test (76.7 vs. 60%, OR = 2.146 [1.319–3.652], *p* = 0.001). ICU admission was significantly more frequent in PCR positive cases compared to negative ones (8.1 vs. 2.6%, OR = 3.379 [1.374–7.048], *p* = 0.010). As regards ICU admitted cases, a higher proportion of male participants was found in the confirmed group (85.7 vs. 57.0%, OR = 0.248 [0.009–1.592], *p* = 0.158). Crude mortality among the confirmed COVID-19 cases was marginally higher than of the PCR negative group (4.7 vs. 1.6%, OR = 3.287 [0.957–8.291], *p* = 0.057).

All patients had symptoms at the time of the first testing among the PCR-positive participants, who later developed severe outcomes and were admitted to the ICU (*n* = 7). As regards deceased patients (*n* = 4), the proportion of initially symptomatic cases was also 100%. We found that among initially asymptomatic patients, no ICU admission or death occurred.

The mean age of participants admitted to the ICU was higher compared to those who did not require intensive care. 85.7% of ICU-admitted patients were male. The mean age of deceased participants was higher compared to those who survived. Three of the four deceased patients were male (Figure 3).

### Testing Results of the General Population and Health Care Providers

**Figure 4:**
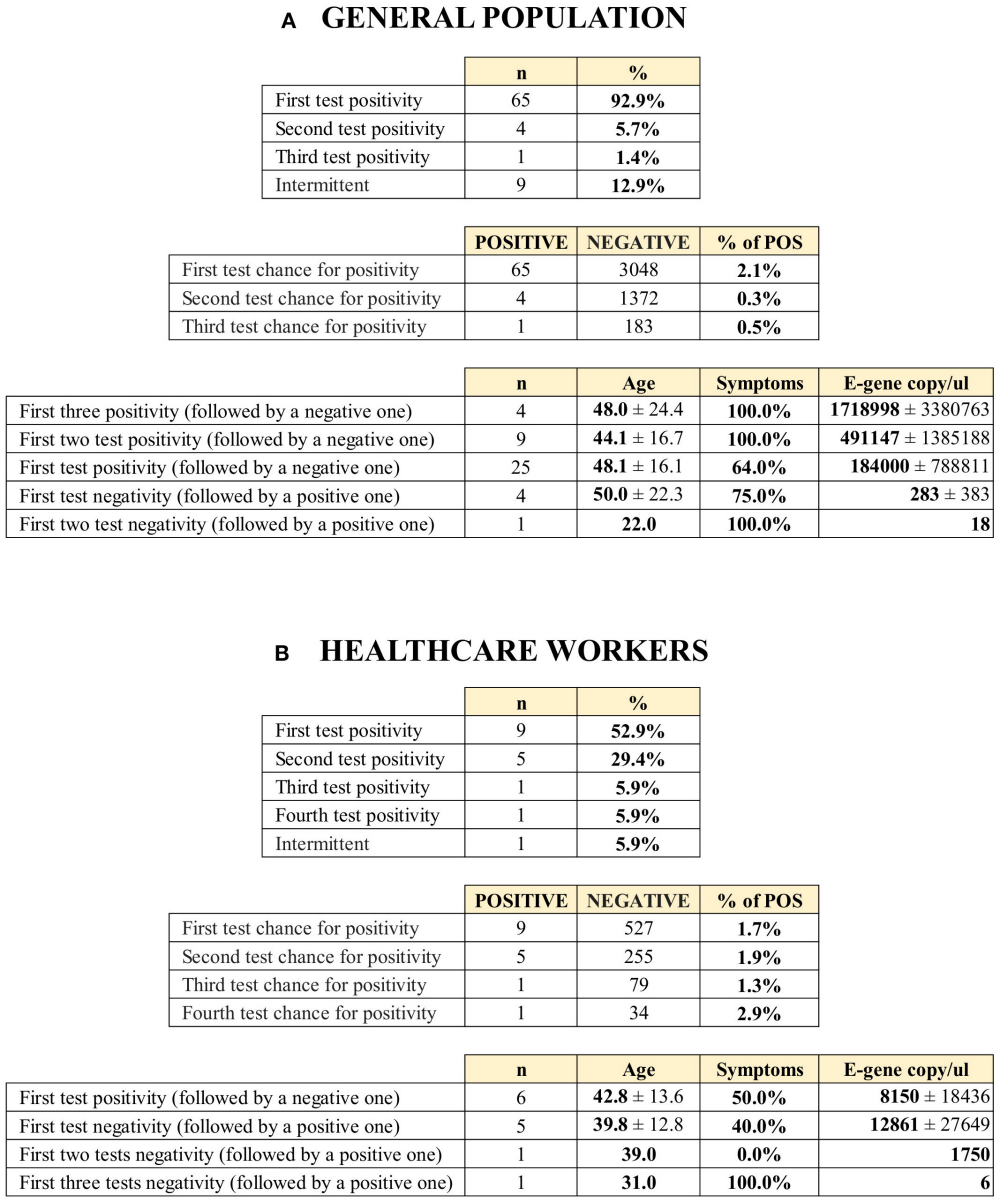
Links between polymerase chain reaction (PCR) positivity with first and subsequent testing and viral excretion among the **(A)** general population and **(B)** health care providers.

As the indication of RT-qPCR testing differs between the general population and health care providers, we also analyzed these two populations separately. During the observation period, we identified 70 and 16 SARS-CoV-2-infected individuals in the general population and among health care providers, respectively (Figure 4; Supplementary Figure 2).

In the general population, the proportion of symptomatic COVID-19 cases was 82.9%. Symptomatic COVID-19 cases had higher viral excretion (copy number of the E-gene) compared to those with asymptomatic infection, although the difference was not statistically significant (328,694 ± 1,198,275 [*n* = 58] vs. 40,234 ± 110,046 [*n* = 12]; W = 257, *p* = 0.159). The rate of cases that tested positive decreased with subsequent testing (2.1, 0.3, and 0.5% during the first, second, and third test, respectively). 92.9% of the positive participants tested positive on their first PCR test. Only 7.1% of the RT-qPCR positive cases (*n* = 5) were identified after a negative first test. Four infections (5.7%) were confirmed with a second test, and only one (1.4%) was confirmed with a third. The viral excretion of the infections identified later was significantly lower compared to those with positive first tests (230 ± 353 vs. 300,707 ± 1,134,581; W = 249, *p* = 0.0499).

Among health care providers, the proportion of symptomatic COVID-19 cases was lower (50.0%). We did not find a difference between symptomatic and asymptomatic health care providers as regards viral excretion (8,901 ± 18,103 vs. 11,637 ± 22,711, respectively; W = 10, *p* = 1). 56.3% of the participants tested positive with the first RT-qPCR test among health care professionals with confirmed infection. Contrary to the general population, the viral excretion of infections identified later was not different from those with positive first tests (10,117 ± 18,957 vs. 9,437 ± 23,326; W = 33, *p* = 0.916) among health care providers.

Absolute quantification of the SARS-CoV-2 genetic segments also allowed us to characterize the individual disease progression with copy number changes. E-gene copy numbers intraindividually showed a decreasing tendency parallel with the relief of clinical symptoms. Patients with higher viral excretion tend to have a longer period of RT-qPCR positivity (Supplementary Figure 3). Remission of the first wave of the local outbreak was observed in close conjunction with a decreasing frequency of the laboratory identification of individuals with high E-gene copy number.

## Discussion

The coronavirus pandemic is spreading progressively. The number of new cases diagnosed daily is still continuously increasing worldwide. There are countries that have engaged in an ongoing fight from the start, and there are countries over the second wave of the pandemic. Even local interception of the fast transmission has a significant impact on the economic and health care burden. Analysis and interpretation of the early results of different local outbreaks should not be delayed because analytical learning is essential to developing effective prevention.

Importantly, if testing capacity becomes insufficient, countries might need to prioritize who is tested (19). Under these circumstances, the WHO recommends that tests be provided for patients at higher risk for developing a more severe disease, for first symptomatic patients in closed communities and for healthcare workers (14). However, we have no evidence on the indication for retesting. Considering this question, our study suggests other factors that would call for further research and recommends their inclusion be considered in future guidelines.

In our study, the rate of cases tested positive decreased with subsequent (second and third) testing; therefore, the number of unnecessary repeat tests was high. This implies that if the number of tests is limited, instead of retesting, resources should be devoted to screening for other suspected COVID-19 cases where the chances of test positivity are higher (20, 21). For example, the presence of COVID-specific symptoms is an important factor to consider (21). This is consistent with our results, as the proportion of PCR-positive cases was higher among participants with symptoms.

There is also no consensus on the timing of the follow-up test. Local protocols mainly determine it by the time of symptom onset and resolution (12). However, the duration of symptoms and viral shedding is not always in synchrony; other factors might therefore also be considered. In our study, higher initial viral loads (E-gene copy numbers) were associated with longer test positivity. This phenomenon has been described by Wolfel et al. (22). They found that higher E-gene copy numbers were associated with a more severe disease course, and the viral load persisted longer compared to those who had lower copy numbers (80 vs. 11 days). Therefore, retesting of patients with high viral loads might be delayed, and the number of unnecessary tests performed too early can be reduced among patients with a high viral load.

In our study, cases with lower initial viral loads turned negative earlier. The isolation and hospitalization could thus end sooner for these patients, leading to economic benefits for both individuals and societies. Lifting the quarantine sooner for these patients can lead to decreased loss of daily wage earnings and reduced isolation costs. In summary, in the case of a PCR test, it is worth considering not only the fact of positivity, but also the degree of viral load.

In addition to obvious infection control aspects, the importance of identifying new cases is supported by the threefold increase of ICU admission and mortality rates among the COVID-19 positive cases in our study.

These results are likely to be independent of demographic features, since they were similar among positive and negative groups, except for the slightly higher rate of male participants among the positive cases. A gender difference favoring men has been observed in previous studies and suggests that the virus is more likely to infect men (23, 24).

Among our limited number of positive cases, elderly, men and symptomatic patients were more likely to be admitted to the ICU or to die in our study, a finding which is consistent with previous results (25).

Previous studies have implied that the vast majority of asymptomatic patients at the time of the first positive test recover spontaneously with a mild disease course (26, 27). We came to the same conclusion since we found that no ICU admission or death occurred among initially asymptomatic patients. These patients thus do not require close observation, and the number of follow-up examinations could therefore be minimized.

The literature suggests that asymptomatic patients can also transmit the disease and viral excretion may be associated with symptomatic patients (28, 29). In contrast, we found much lower E-gene copy numbers in the asymptomatic cases emerging from the general population compared to the symptomatic ones.

It is possible that some of these patients were pre-symptomatic at the time of testing and developed symptoms later. A study comparing truly asymptomatic and pre-symptomatic cases found that the virus could be detected for a longer period in pre-symptomatic cases (26, 30). If we add this to our findings of gene copy number and duration of test positivity, truly asymptomatic patients may be candidates for earlier retesting and released from isolation as soon as possible.

Healthcare personnel were analyzed separately because the PCR test indication was fundamentally different in their case, and this population has different demographic characteristics. In their case, our previous findings are not necessarily correct. Nevertheless, it is difficult to draw a definite conclusion because of the small sample size. We hypothesize that these individuals may have been identified at an earlier stage of the disease. Based on a previous study, we can also assume that they were exposed to a lower viral load due to the use of protective equipment, resulting in lower viral gene concentration in their samples (31).

### Strength and Limitations

This is the first multi-center study in Hungary that reports on the links between PCR testing and viral excretion, along with demographic and clinical data. The observation period covers the entire first wave of the region under pandemic surveillance of the COVID-19 outbreak, and we included every sample of suspected cases analyzed in the primary testing center Hungary's Southern Transdanubian region (32). Nevertheless, virus isolation was mostly performed manually, which allows the detection of lower virus copy numbers.

This study has some limitations. The first is the retrospective nature of the data collection. Secondly, despite the large number of tests performed, our conclusions may be limited by the relatively low number of confirmed cases and its influence on the power of the performed statistical analyses. Lastly, some deviations occurred in distant areas following the strict screening protocol in some cases, which resulted in missing data.

Although, most of our results are in line with exiting published data, these new data from the specified Hungarian population contribute to the knowledge and understanding of this global pandemic.

### Implication for Practice

- To avoid diagnostic insufficiency, when testing capacity reaches its limits in the future, focusing on testing new cases instead of repeated screening could be feasible.
- We recommend considering the viral copy number when choosing the timing for retesting positive cases (follow-up tests). Our results support earlier follow-up testing with lower gene copy numbers and delayed follow-up testing with higher copy numbers.
- Quantitative detection of viral excretion and different segments of the viral genome which help to determine a potential infectious state may be useful for clinicians to plan patient management, placement in the proper health care ward and translocation.
- We would like to draw clinicians' attention to an important finding: mortality and ICU admission were three times more common among confirmed cases compared to “only” suspected cases; however, further analyses are required with larger datasets, as the difference was not significant due to the low positive case numbers.
- Lack of symptoms at the time of the first test indicates a good outcome.

### Implications for Research

Additional studies are warranted to confirm our recommendations. A particularly important area of research is the relation between viral load and disease duration. Further studies need to identify factors that can narrow the range of testing indication in the case of insufficient testing capacity.

## Data Availability Statement

The raw data supporting the conclusions of this article will be made available by the authors, without undue reservation.

## Ethics Statement

The studies involving human participants were reviewed and approved by National Center for Public Health (20800-6/2020/EÜIG). Written informed consent from the participants' legal guardian/next of kin was not required to participate in this study in accordance with the national legislation and the institutional requirements.

## Author Contributions

KG, PH, and AS: conceptualization. KG, LG, DC, KF, TN, and BS: data collection and curation. KG, RH, and AS: formal analysis. LG, DC, and PH: funding acquisition. KG, AG, TN, AM, PH, and AS: methodology. AG, AM, and PH: resources. AG, PH, and AS: supervision. RH and AS: visualization. KG, MF, SK, and PH: writing – original draft. KG, MF, SK, RH, AG, LG, DC, KF, TN, AM, BS, PH, and AS: writing – review & editing. All authors contributed to the article and approved the submitted version.

## Conflict of Interest

The authors declare that the research was conducted in the absence of any commercial or financial relationships that could be construed as a potential conflict of interest.

## Supplementary Material

Supplementary Figure 1 The algorithm for SARS-CoV-2 polymerase chain reaction (PCR) testing.

Supplementary Figure 2 Polymerase chain reaction (PCR) results, viral excretion and presence of symptoms with first test among the **(A)** general population and **(B)** health care providers. SP, symptomatic positive; NSP, non-symptomatic positive; SN, symptomatic negative; NSN, non-symptomatic negative.

Supplementary Figure 3 Results of individual disease progression with E-gene copy number changes.

Supplementary Document 1 Details of the assessment of viral excretion, sample processing, PCR amplification, and mapping methodology.

Supplementary Table 1 **(A)** Median, quartiles, minimum and maximum values in the negative and positive severity and mortality groups. **(B)** Sex distribution, symptoms, severity and mortality among positive cases in the normal population and healthcare personnel groups. **(C)** Sex distribution, symptoms, severity and mortality in the negative and positive groups. **(D)** First, second and third test positivity in the normal population.
