# Supplementary material for: Analysis of COVID-19-Related RT-qPCR Test Results in Hungary: Epidemiology, Diagnostics, and Clinical Outcome
Source: Front Med (Lausanne). 2021 Jan 26;7:625673. doi: 10.3389/fmed.2020.625673 (PMC7870862; doi:10.3389/fmed.2020.625673)

The algorithm for SARS-CoV-2 polymerase chain reaction (PCR) testing

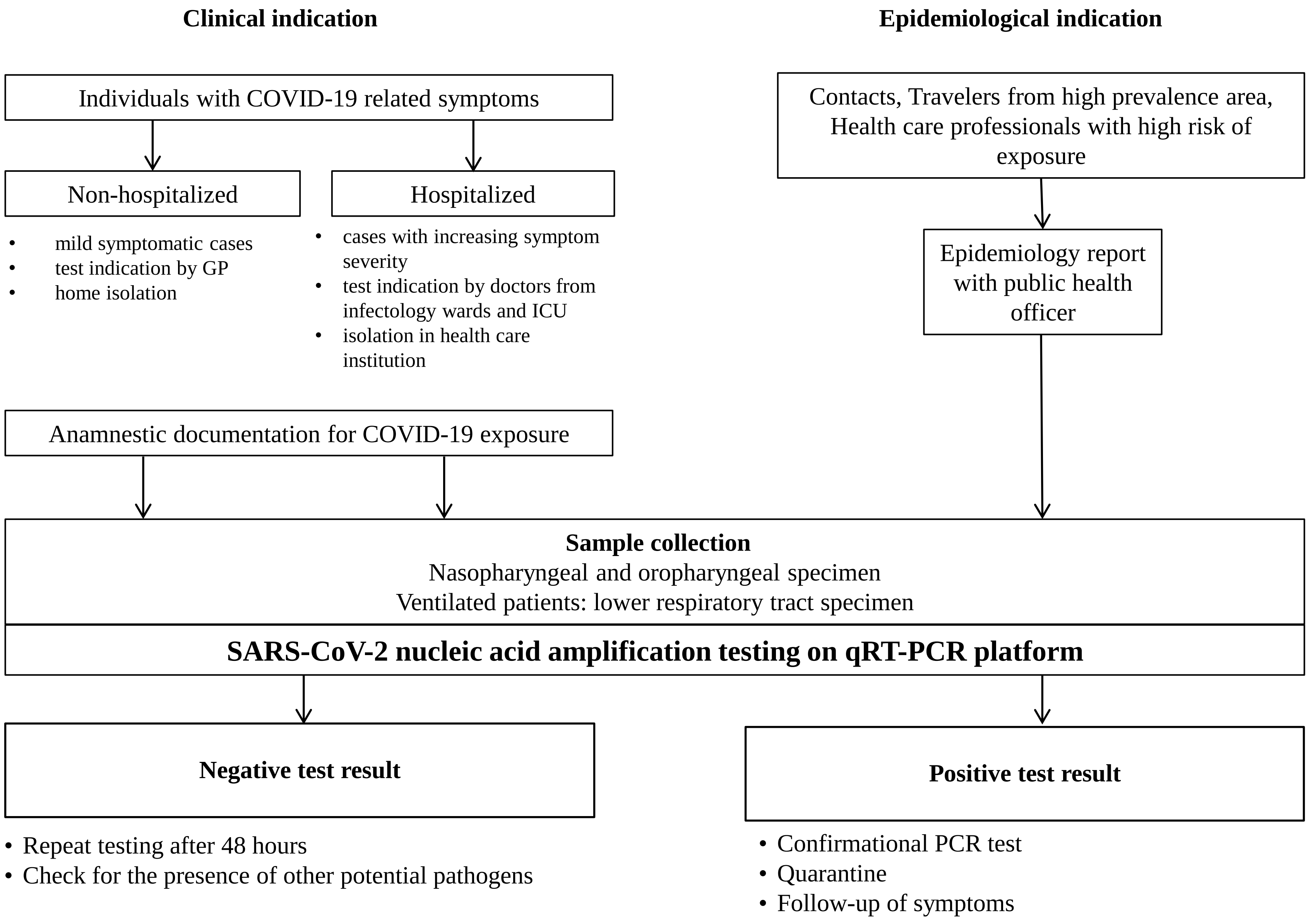

Supplement: Supplementary Figure 1 — The algorithm for SARS-CoV-2 polymerase chain reaction (PCR) testing. [file Data_Sheet_2.PDF]
