# Supplementary material for: Analysis of COVID-19-Related RT-qPCR Test Results in Hungary: Epidemiology, Diagnostics, and Clinical Outcome
Source: Front Med (Lausanne). 2021 Jan 26;7:625673. doi: 10.3389/fmed.2020.625673 (PMC7870862; doi:10.3389/fmed.2020.625673)
Supplement: Supplementary Figure 2 — Polymerase chain reaction (PCR) results, viral excretion and presence of symptoms with first test among the (A) general population and (B) health care providers. SP, symptomatic positive; NSP, non-symptomatic positive; SN, symptomatic negative; NSN, non-symptomatic negative. [file Data_Sheet_4.PDF]

A) GENERAL POPULATION

Age	E gene copy/ul	Symptoms	Number of tests												
			1	2	3	4	5	6	7	8	9	10	11	12	13
26	9800	SP	positive	positive	positive	positive	negative	positive	negative	negative	negative	positive	negative	negative	negative
27	23500	SP	positive	positive	positive	negative	positive	positive	negative	negative	negative				
79	493	SP	positive	positive	positive	negative	positive	negative	negative						
30	62000	SP	positive	positive	positive	negative	positive	negative	negative						
56	6790000	SP	positive	positive	positive	negative	negative	negative							
59	477	SP	positive	positive	positive										
68	3510	SP	positive	positive	negative	positive	negative	negative	negative						
41	1	SP	positive	positive	negative	negative	negative								
63	13	SP	positive	positive	negative	negative	negative								
28	4180000	SP	positive	positive	negative	negative									
23	120	SP	positive	positive	negative	negative									
27	2040	SP	positive	positive	negative	negative									
56	844	SP	positive	positive	negative	negative									
54	220000	SP	positive	positive	negative	negative									
37	13800	SP	positive	positive	negative										
78	2870	SP	positive	positive											
59	28700	SP	positive	positive											
46	86400	SP	positive	positive											
79	83	SP	positive	positive											
68	78000	SP	positive	positive											
61	452	SP	positive	positive											
72	139	SP	positive	negative	positive	negative	negative	positive	negative	negative	negative				
41	3950000	SP	positive	negative	positive	negative	negative								
26	1470	SP	positive	negative	negative	positive	negative	negative	negative						
46	1390	NSP	positive	negative	negative	positive	negative	negative							
81	83800	SP	positive	negative	negative	negative									
64	9250	SP	positive	negative	negative	negative									
39	169	SP	positive	negative	negative	negative									
38	16	SP	positive	negative	negative	negative									
80	820	NSP	positive	negative	negative										
40	4	NSP	positive	negative	negative										
38	165000	SP	positive	negative	negative										
48	1300	SP	positive	negative	negative										
70	263	SP	positive	negative	negative										
28	1890	SP	positive	negative	negative										
54	15	SP	positive	negative	negative										
41	2960	NSP	positive	negative	negative										
23	378000	NSP	positive	negative											
62	1300	SP	positive	negative											
29	34	NSP	positive	negative											
49	13	NSP	positive	negative											
46	782	SP	positive	negative											
59	947	SP	positive	negative											
39	84	NSP	positive	negative											
50	346	SP	positive	negative											
40	17	NSP	positive	negative											
34	642	SP	positive												
76	35	SP	positive												
74	17	SP	positive												
77	34200	SP	positive												
71	1001	SP	positive												
57	76	SP	positive												
18	98700	NSP	positive												
41	7	SP	positive												
72	18	SP	positive												
68	2960000	SP	positive												
78	173	SP	positive												
74	6260	SP	positive												
31	250000	SP	positive												
42	780	NSP	positive												
37	8	SP	positive												
87	64500	SP	positive												
44	7850	SP	positive												
57	4570	SP	positive												
60	14000	SP	positive												
55	54	SN	negative	positive	positive	negative	negative								
79	12	NSN	negative	positive											
28	225	SN	negative	positive											
38	842	SN	negative	positive											
22	18	SN	negative	negative	positive	negative	negative								

negative
positive

B) HEALTHCARE WORKERS

Age	E gene copy/ul	Symptoms	Number of tests												
			1	2	3	4	5	6	7	8	9	10	11	12	13
63	169	SP	positive	positive											
52	3100	SP	positive	negative	positive	negative	negative								
41	5	SP	positive	negative	negative	negative									
34	45700	NSP	positive	negative	negative										
53	4	NSP	positive	negative	negative										
56	32	SP	positive	negative	negative										
21	64	NSP	positive	negative	negative										
61	41200	SP	positive												
62	782	NSP	positive												
50	1930	NSN	negative	positive	positive	positive	negative	negative							
29	63	NSN	negative	positive	negative	negative									
37	6	SN	negative	positive	negative	negative									
56	62300	NSN	negative	positive	negative	negative									
27	6	SN	negative	positive	negative										
39	1750	NSN	negative	negative	positive	positive	negative	negative							
31	6	SN	negative	negative	negative	positive	negative	negative	negative						

negative
positive
