# Supplementary figures and images for: Analysis of COVID-19-Related RT-qPCR Test Results in Hungary: Epidemiology, Diagnostics, and Clinical Outcome
Source: Front Med (Lausanne). 2021 Jan 26;7:625673. doi: 10.3389/fmed.2020.625673 (PMC7870862; doi:10.3389/fmed.2020.625673)

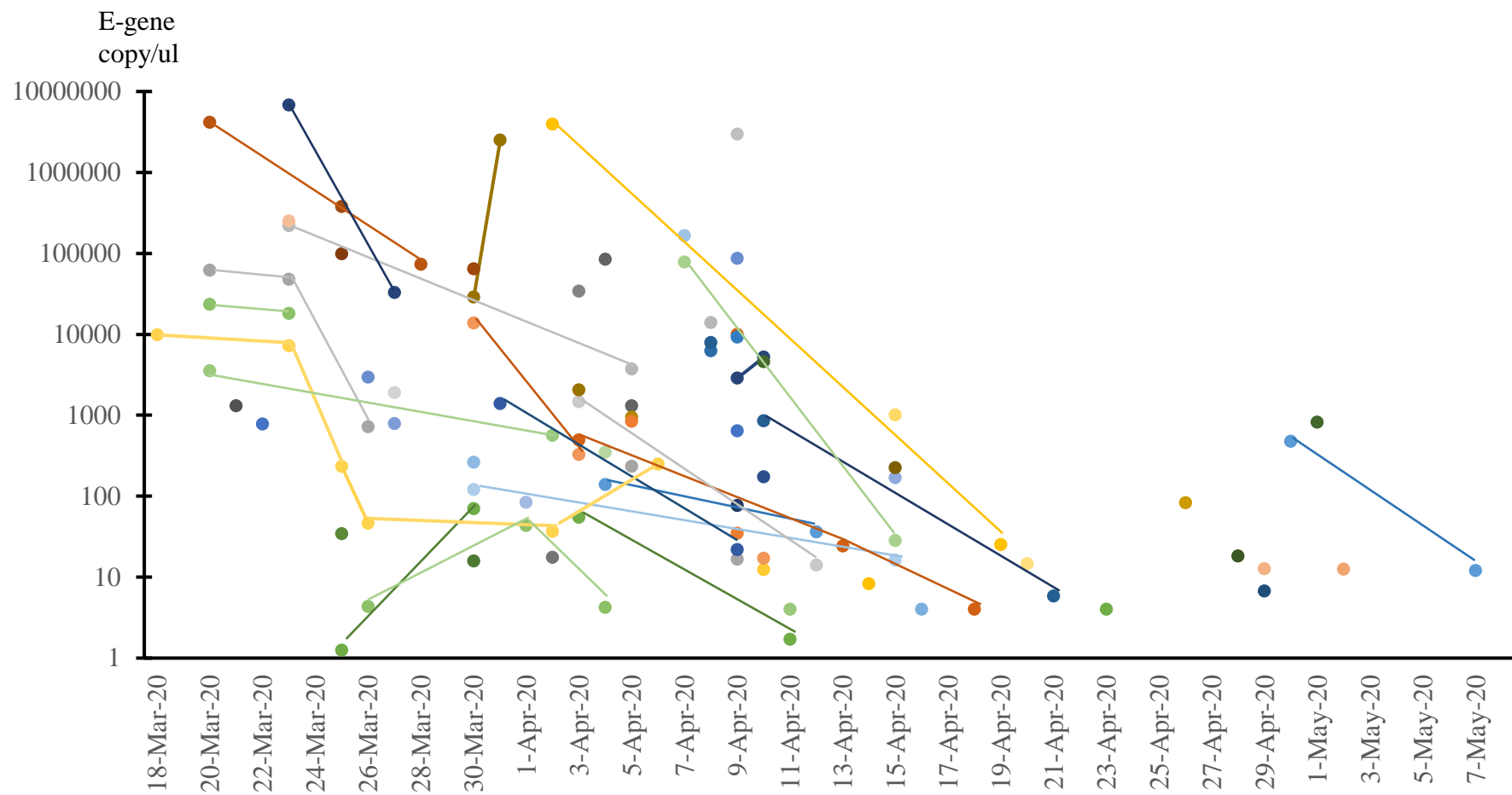

Supplement: Supplementary Figure 3 — Results of individual disease progression with E-gene copy number changes. [file Data_Sheet_5.PDF]
