# Supplementary material for: Analysis of COVID-19-Related RT-qPCR Test Results in Hungary: Epidemiology, Diagnostics, and Clinical Outcome
Source: Front Med (Lausanne). 2021 Jan 26;7:625673. doi: 10.3389/fmed.2020.625673 (PMC7870862; doi:10.3389/fmed.2020.625673)
Supplement: Supplementary Document 1 — Details of the assessment of viral excretion, sample processing, PCR amplification, and mapping methodology. [file Data_Sheet_1.PDF]

Details of the assessment of viral excretion, sample processing, PCR amplification and mapping methodology.

Sample collection

Samples were taken by health care professionals of the National Emergency Service or of the local health care providers from the upper respiratory tract (nasopharyngeal and oropharyngeal swabs) and/or lower respiratory tract (tracheal sputum) specimen in hospitalized and ventilated cases.

Sample processing, PCR amplification

According to the standard sampling, swab was washed into the sample collection tube containing virus transport medium (CE certified, Biolabs Ltd, Hungary) and safely discarded. Sample collection tubes were individually wrapped in sterile double wall plastic bag and transferred to the laboratory at 4 °C for nucleic acid extraction. Nucleic acid was extracted from 200µl specimen either manually or with the MagNaPure 96 automated nucleic acid extraction system (Roche, Mannheim, Germany). Automated extraction was optimized using the MagNA Pure 96 DNA and Viral NA SV Kit (Cat No. 654358800, Roche) according to the manufacturer's instructions. During manual nucleic acid extraction HighPure RNA isolation kit (Cat No.11858882001, Roche) and provided protocol was followed. Both automated and manual nucleic acid extraction procedure included LightMix Modular EAV RNA extraction control (Cat No. 61090996, TIB Molbiol) to verify extraction and reverse transcription. 5µl of the extracted RNA was used for rRT-qPCR analysis to detect the presence of SARS-CoV-2 RNA targeting three regions of the positive-sense single stranded viral genome: conserved fragments of the sequences encoding the envelope protein (E-gene), neurocapsid protein (N-gene) and RNA dependent RNA polymerase (RdRP-gene). LightMix Modular SARS-CoV (COVID19) E-gene (Cat No. 53077696), LightMix Modular SARS-CoV (COVID19) N-gene (Cat No. 53077596) and LightMix Modular SARS-CoV (COVID19) RdRP (Cat No. 53077796) were used combined with the LightMix Modular EAV RNA extraction control 610 for multiplex PCR target and extraction control detection. PCR Master mixes were prepared to contain 0.5 µl of target specific primer and probe mix, 0.5 µl extraction control target specific primer and probe mix, 4 µl of Real Time ready Virus Master reaction buffer and 0.4 µl Real Time ready Virus Master RT enzyme (Cat No. 05992877001, Roche), 10.4 µl PCR grade water and 5 µl of the RNA sample. For negative controls we prepared a mastermix where template RNA was substituted with PCR grade water. PCR amplification was carried out in LightCycler 480 and Cobas Z 480 PCR systems with the following thermic conditions (reverse transcription 1 cycle: 55°C for 5 min; enzyme activation 1 cycle: 95 °C for 5 min; amplification 45 cycles {95 °C for 5s, 60 °C for 15s, 72 °C for 15s}. Results were analysed and fluorescence data was converted into concentrations using standard curve. Standard curve was generated on quantitative real-time PCR, based on the dilution series of the positive control provided with the LightMix Modular E, N and RdRP kits, which were previously quantitatively analyzed using droplet digital PCR system and BioRad QX200 PCR dd platformand ddPCR Expert Design Assay: 2019-nCoV CDC ddPCR Triplex Probe Assay.

Generation of the database

Based upon the revision of submitted questionnaire, rRT-qPCR results and hospitalized patient's medical records we generated a database and listed 5463 tested cases between 17th of March and 8th of May. Besides PCR test results for SARS-CoV-2 positivity we collected epidemiological, demographic, clinical, and outcome data: reason of testing, date of sampling, age, gender, presence of symptoms, viral excretion, ICU admission, and mortality. Absolute quantification analysis results of the SARS-CoV-2 E-gene, N-gene and RdRP-gene targets detected during the epidemic period were plotted according to timeline and the repeated tests. Positive and negative test results were required to be confirmed with

two PCR tests performed from two separate sampling when swabs were taken with at least 48-hour difference. Hospitalized patients who were treated in the Clinical Center of Pécs University were followed closely and in these cases samples were taken according to presence and changes of their symptoms. Positive cases identified in Zala County were confirmed by the National Public Health Center's Reference Laboratory, these test results were not included to the database.

Methodology of creating maps showing the incident cases representing the dynamics of the epidemic spreading (Figure 2)

Positive cases were marked on a geographic map of the south transdanubian region according to residence of the recognized subjects and size of the marker dot is proportionate to the number of cases found in the settlement.

Maps were created with the help of MMQGIS (v2020.1.16) and TimeManager (v3.14) plugins from Quantum GIS v3.12 (QGIS 2020). Two vector layers were used to create the maps: Hungarian counties (polygon layer) and the settlements (point layer). Data were reshaped to get the necessary format from which we could create the maps with the help of the above-mentioned plugins.

QGIS.org (2020). QGIS Geographic Information System. Open Source Geospatial Foundation Project. http://qgis.org".
MMQGIS 2020 v2020.1.16 https://plugins.qgis.org/plugins/mmqgis/
TimeManager 2020 v3.14 https://plugins.qgis.org/plugins/timemanager/
