# Supplementary material for: Analysis of COVID-19-Related RT-qPCR Test Results in Hungary: Epidemiology, Diagnostics, and Clinical Outcome
Source: Front Med (Lausanne). 2021 Jan 26;7:625673. doi: 10.3389/fmed.2020.625673 (PMC7870862; doi:10.3389/fmed.2020.625673)
Supplement: Supplementary Table 1 — (A) Median, quartiles, minimum and maximum values in the negative and positive severity and mortality groups. (B) Sex distribution, symptoms, severity and mortality among positive cases in the normal population and healthcare personnel groups. (C) Sex distribution, symptoms, severity and mortality in the negative and positive groups. (D) First, second and third test positivity in the normal population. [file Data_Sheet_3.PDF]

Supplementary Table 1.A. Median, quartiles, minimum and maximum values in the negative and positive severity and mortality groups.

	Negative	Negative Mild	Negative ICU	Negative Mort	Positive	Positive Mild	Positive ICU	Positive Mort
Min	0,0	0,0	0,0	29,0	18,0	18,0	61,0	61,0
Q1	35,0	35,0	54,0	63,0	37,0	35,5	63,5	63,3
Median	52,0	52,0	64,0	71,5	49,5	46,0	68,0	68,0
Q3	69,0	68,0	75,0	79,0	62,8	59,5	70,0	75,8
Max	98,0	98,0	91,0	96,0	87,0	81,0	87,0	87,0
IQR	34,0	33,0	21,0	16,0	25,8	24,0	6,5	12,5
Upper Outliers	0,0	0,0	0,0	0,0	0,0	0,0	1,0	0,0
Lower Outliers	0,0	0,0	7,0	2,0	0,0	0,0	0,0	0,0

Supplementary Table 1.B. . Sex distribution, symptoms, severity and mortality among positive cases in the normal population and healthcare personnel groups.

	men	women	non-symp	symp	ICU	non-ICU	mortality	no mortality
normal population	41	29	12	58	7	63	4	66
healthcare pers	6	10	8	8	0	16	0	16

Supplementary Table 1.C. Sex distribution, symptoms, severity and mortality in the negative and positive groups.

	men	women	non-symp	symp	ICU	non-ICU	mortality	no mortality
Negative	1696	1875	1411	2157	93	3478	54	3517
Positive	46	40	20	66	7	79	4	82

Supplementary Table 1.D. First, second and third test positivity in the normal population.

	1st test	2nd and 3rd test	Total
Negative	3048	1555	4603
Positive	65	5	70
Total	3113	1560	4673

healthcare pers: healthcare personnel; ICU: intensive care unit admission; mort: mortality; non-symp: non-symptomatic; symp: symptomatic
